# Multimode Design and Piezoelectric Substrate Anisotropy Use to Improve Performance of Acoustic Liquid Sensors

**DOI:** 10.3390/s22197231

**Published:** 2022-09-23

**Authors:** Andrey Smirnov, Vladimir Anisimkin, Natalia Voronova, Elizaveta Shamsutdinova, Peng Li, Hamdi Ezzin, Zhenghua Qian, Tingfeng Ma, Iren Kuznetsova

**Affiliations:** 1 Kotelnikov Institute of Radio Engineering and Electronics of RAS, 125009 Moscow, Russia; 2 Acoustoelectronic and Piezokeramic ELPA Corporation, 124460 Moscow, Russia; 3 State Key Laboratory of Mechanics and Control of Mechanical Structures, College of Aerospace Engineering, Nanjing University of Aeronautic and Astronautic, Nanjing 210016, China; 4 Piezoelectric Device Laboratory, School of Mechanical Engineering and Mechanics, Ningbo University, Ningbo 315211, China

**Keywords:** plate acoustic waves, liquid sensor, substrate anisotropy, liquid conductivity

## Abstract

Using acoustic wave modes propagation in piezoelectric plates loaded with conductive liquids, peculiarities of the mode-liquid acoustoelectric interaction are studied. It is found that (i) in contrast to bulk and surface acoustic waves propagating in piezoelectric semiconductors, the acoustoelectric attenuation of the modes is not symmetric in respect to its maximum, (ii) a large increase in attenuation may be accompanied by a small decrease in phase velocity and vice versa, (iii) the peculiarities are valid for “pure” (without beam steering) and “not pure” (with beam steering) modes, as well as for modes of different orders and polarizations, and (iv) conductivity of test liquid increases electromagnetic leakage between input and output transducers, affecting results of the measurements. To decrease the leakage, the liquid should be localized between transducers, outside the zone over them. If so, the mode sensitivity may be as large as 8.6 dB/(S/m) for amplitude and 107°/(S/m) for phase. However, because of comparable cross-sensitivity towards viscosity and dielectric permittivity, modes with selective detection of liquid conductivity are not found.

## 1. Introduction

It is well-known that a change in electric conductivity of an adjacent medium located on a surface of a piezoelectric substrate produces a change in phase velocity and attenuation of any acoustic wave with nonzero coupling constant (Rayleigh [1,2,3], Love [3], Lamb [4,5], zero-order shear-horizontal [6,7], higher-order [8], slot [9,10], etc.). For most waves, the phase velocity decreases with conductivity monotonically, approaching its minimum value when tangential components of electric fields accompanying the wave are completely shorted [1,2,3,4,5,6,7,8,9,10]. On the other hand, for particular cases of slightly inhomogeneous piezoactive surface acoustic waves with shear-horizontal polarization (Bleustein-Gulyaev and/or Love waves), an increase in conductivity of a substrate or conductive layer deposited on the substrate produces, first, an increase in phase velocity of the wave until some maximal value and then, a decrease in the velocity to some minimal value [11,12]. This property originates from the fact that for small conductivities, a change in electrical boundary conditions enhances localization of the wave electric fields near the interface and, thereby, enlarges the wave velocity in a propagation medium. The effect of the field localization is larger than the effect of screening the tangential electric wave components up to a certain conductivity. However, when the conductivity is large enough, the screening effect becomes dominant and the phase velocity of the wave behaves in a common manner, monotonically decreasing [11,12].

The acoustoelectric attenuation behaves similar for all types of acoustic waves: it increases for small conductivities, approaches some maximum for intermediate conductivities, and decreases to zero for large conductivities of the substrate or layer [1,2,3,4,5,6,7,8,9,10,11,12]. This property is due to the fact that at low conductivities, the layer behaves like dielectric, while at high conductivities it is more like an ideal conductor. For intermediate conductivities, the structure is characterized by a certain Maxwellian relaxation frequency f_M_ = σ_S_/ε_eff_, where σ_S_ is the surface conductivity of the layer, and ε_eff_ is the effective permittivity depending on σ_S_, plate thickness, and wavelength. When acoustic wave frequency f becomes equal to the Maxwellian relaxation frequency f_M_ (f = f_M_), a resonant interaction between an acoustic wave and a structure takes place and the acoustoelectric attenuation approaches maximum [4,7]. It should be noted that for a substrate coated with a conductive solid layer, the dependence of the attenuation on the surface conductivity is symmetrical. On the contrary, the same dependence for a zero-order shear-horizontal wave in a plate coated with a conductive *liquid* layer is turned to be asymmetrical [13].

The dependence of acoustic wave properties on a liquid conductivity made it possible to propose a large number of acoustoelectronic sensors. First, of them were based on shear-horizontal surface acoustic waves characterized by maximal value of the mechanical displacement component lying in the plane perpendicular to the wave vector [14,15,16,17]. Further, it was suggested to use for the same sensors the zero-order waves with shear-horizontal polarization propagating in the thin plate whose thickness is comparable with the acoustic wavelength. The waves of this type are characterized by the largest electromechanical coupling coefficients among all other waves in the same materials [7,13,18]. Furthermore, the spectrum of higher-order waves in piezoelectric plates with a thickness comparable to or greater than the wavelength is very wide and the number and properties of the plate waves are purposefully varied by the plate thickness *h*, wavelength *λ*, mode order *n*, and propagation direction [19,20,21,22,23,24,25,26]. Electric fields of the modes penetrate into contacting liquid and interact with free charge carriers. This interaction results in a change in the phase velocities *v* (phases *ϕ*) and amplitudes (attenuation *α*) of the waves. Although the acoustoelectronic interaction takes place only in a thin liquid layer (~10 μm) adjacent to the interface and only in a narrow range of liquid conductivities (*σ* ≈ 0.01 ÷ 10 S/m [7]), first-order plate modes have proven to be useful for applications such as the detection of yeasts and bacteria [27,28] and registration of the water-ice phase transition [20,29]. The propagation of shear-horizontal waves in the conductive liquid-loaded functionally graded porous piezoelectric media (FGPPM) has been investigated recently. The dispersion and the attenuation curves have been plotted to describe the impact of various parameters, namely gradient parameter of both the piezoelectric material, thickness of FGPPM plate, imperfect interface parameter, relative permittivity, and conductivity of the liquid medium [30]. A thickness shear solidly mounted resonator based on Yttrium-doped AlN has been suggested recently as a high-sensitive liquid sensor [31]. A lateral electric field-excited resonator based on piezoceramic has also been suggested as a sensor for liquid conductivity measurements [32].

Attractive property of the acoustic plate waves is also the change in the power flow direction through the change in the electrical boundary conditions and propagation direction. This property, originated from anisotropy of the plate, has already been demonstrated in [33,34].

In conclusion, expansion of the acoustic plate wave applications requires the study of acoustoelectronic interaction in a more general case for the whole spectrum of the waves including modes of high orders and modes with beam steering. It is also interesting to estimate achievable sensitivity of the wave towards electric conductivity and compare it with those for liquid viscosity and dielectric permittivity.

The purpose of this paper is the experimental studying of the effect of liquid conductivity on the characteristics of the higher-order waves in piezoelectric plates, as well as the use of piezoelectric plate anisotropy to improve the performance of the liquid sensors.

## 2. Materials and Methods

**Figure 1:**
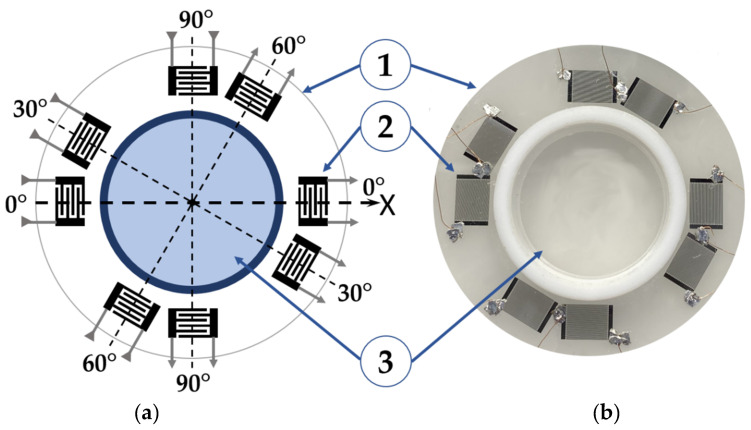
Schematic view (**a**) and photo (**b**) of experimental sample with 4 acoustic delay lines arranged on same piezoelectric plate; 1—plate; 2—interdigital transducers (IDTs); 3—liquid cell. Acoustic plate modes propagate in the plate at angles 0°, 30°, 60°, and 90° off X axis.

Schematic view of a test sample is shown in Figure 1. It consists of 4 delay lines with input and output interdigital transducers (IDTs) arranged on same piezoelectric plate at different angles (Θ = 0°, 30°, 60°, 90°) with respect to crystallographic axis X (Table 1). The number of acoustic plate modes generated in each line is about 10–15. The total amount of modes detected in the plate is 40–60. Properties of all modes are different. They are varied with mode order *n*, propagation direction (angle Θ), material of the plate, plate thickness *h*, and wavelength *λ* (period of the transducers).

The plate materials used in experiments are commercially available 128Y-*LiNbO*_3_ (Euler angles 0°, 37.86°, Θ), 41Y- *LiNbO*_3_ (0°, −49°, Θ), Y-*LiNbO*_3_ (0°, 90°, Θ), and 36Y-*LiTaO*_3_ (0°, −54°, Θ) with thickness *h* = 350 and 500 μm. The plates have one grinded and one polished surface. The grinded surface (optical class Δ10) has averaged horizontal and vertical roughness 0.16 μm and 0.8 μm, respectively. The polished surface (optical class Δ14) has averaged horizontal and vertical roughness 0.01 μm and 0.05 μm, respectively. The polished surface is coated with liquid cell (teflon) 18 mm in diameter *L* and four pairs of IDTs at (Θ = 0°, 30°, 60°, 90° off X axis), as shown in Figure 1. Volume of the liquid sufficient for the measurements is as small as 100 μL.

Each transducer comprises 20 finger electrodes followed with period *λ* = 200 μm patterned from 100-nanometer thick *Cr* and 1000-nanometer thick *Al*. Large number of electrodes pairs (20) provides narrow pass band of the transducers (5%) and good frequency resolution of the modes (±0.5 MHz) with close velocities *v_n_* (±0.2 × 10^6^ m/s). The normalized plate thickness equal to *h*/*λ* = 350 μm/200 μm = 1.75 and 500 μm/200 μm = 2.5 ensure large number of the modes in each direction and the mode’s variety. Different orientations of the transducers allow to compare “pure” directions (Θ = 0°, 90°), where acoustic beams of all modes are parallel to propagation direction (no beam steering, the power flow angle *Ψ_n_* = 0°), and “not pure” directions (Θ = 30°, 60°), where there is beam steering for all modes (*Ψ_n_* ≠ 0°).

The angle *Ψ_n_* for acoustic plate modes is calculated for other acoustic waves [35]:

*Ψ_n_* = *arctg*(1/*v_n_*) (*dv_n_*/*d*Θ), (1)

where *dv_n_* is a change in a mode velocity due to a change (*d*Θ) in propagation direction, and *Ψ_n_* is an angle between a mode beam and a propagation direction on a plate face. Both Θ and *Ψ_n_* are taken in radians in (1).

Calculations of *Ψ_n_* are accomplished for the first 10 modes (*n =* 0–9), using well-approved software [36] and material constants [35]. In order to calculate orientation dependence *Ψ_n_(Θ)* for each mode, the angle *Θ* between propagation direction and X axis is changed from *Θ* = 0° (+X axis) to *Θ* = 180° (–X axis) with the step 3°, holding other two Euler angles constant. In order to follow a given mode and avoid a skip from one mode to the other, the depth profiles of the modes are controlled. It allows for observing a smooth evolution of any investigated mode during the calculations for different *Θ*. Like for surface and bulk acoustic waves, the as-calculated dependence *Ψ_n_(Θ)* is originated from anisotropy of the crystals, but unlike waves of other types, this dependence for acoustic plate modes is changed also with mode order *n* and plate thickness *h/*λ [37]: for a given mode *n* and angle *Θ,* the angle *Ψ_n_(Θ)* is varied with plate thickness *h/*λ; for a given plate thickness *h/*λ and angle *Θ*, the angle *Ψ_n_(*Θ*)* is varied with mode order *n*.

**Figure 2:**
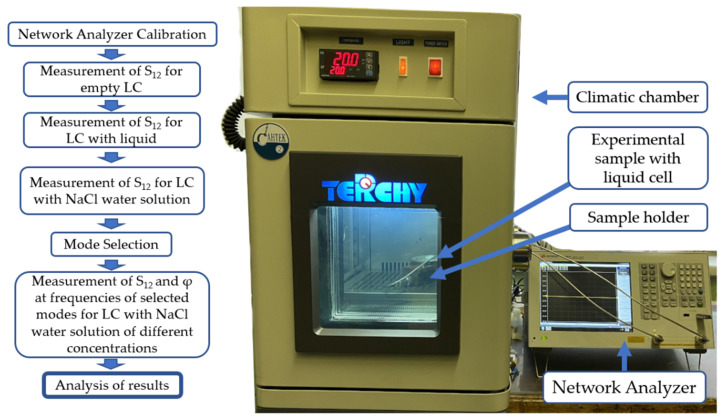
Flow chart of the experiments and schematic view of the experimental setup.

Acoustic delay line is placed in climatic chamber UC-20CE (Terchy Technology LTD, Taiwan, 20l in volume) fixed at *T* = 20 ± 0.1 °C (293.15 K). From the chamber, the sample is connected with KEYSIGHT 5061B network analyzer (Keysight, Santa Rosa, CA, USA) operating in amplitude (*S*_21_) or phase (ϕ_21_) formats. First, insertion loss (transfer function *S*_21_) of the delay line versus frequency *f* is measured without any liquid in the cell (*S*_21_^AIR^). This measurement provides the spectrum of acoustic modes existing in plate at relevant frequencies *f_n_* = *v_n_*/λ. Second, the measurements of *S*_21_ and ϕ_21_ are carried out, when reference liquid (distilled water, 200 mL) and test liquid (see below) are introduced into the cell one by one. It provides modes with best sensitivity towards a test liquid, i.e., the modes with large amplitude response Δ*S*_12_ = *S*_12_ ^Lq^ − *S*_1_^H2O^ and/or large phase response Δϕ_12_ = ϕ_12_^Lq^ − ϕ_12_^H2O^. Finally, the time variations in the amplitude Δ*S*_12_(*t*) and phase Δϕ_12_(*t*) responses are measured for the most sensitive modes when drops of test liquid (40 mL) are induced one by one into the cell with distilled water at regular time intervals Δ*t* ≈ 100 sec. Knowing mass of the drop, drops number, mass of distilled water in the cell, and relevant acoustic responses, calibration curves for a given mode and a given liquid are plotted. Experimental results for different modes, plates, and propagation directions are compared with one another and best of them are tabulated. Precisions of the measurements are ±0.1 dB for *S*_12_ and ±0.1 degree for phase. The corresponding flow chart of the experiments and experimental setup is presented in Figure 2.

*Absolute* values of the phases are not useful for comparing different samples because electric contributions to the phases are changed from sample to sample unpredictably depending on length of wires, quality of contacts, location of sample in holder, etc. Comparison is useful only for *phase responses* Δϕ = ϕ ^Lq^ − ϕ^H2O^, when one and the same electric contributions to ϕ^Lq^ and ϕ^H2O^ mutually compensate one another for each sample.

Distilled water is used as reference liquid because of its low conductivity (*σ* < 10^−3^ S/m) and small viscosity (*η* = 1.03 cP).

Water solutions of NaCl are used as test liquids with variable conductivity changed from *σ* = 0 (distilled water) to *σ* = 10 S/m (7.6 weight % NaCl in water) by changing weight per cent of the solution components. Viscosity (<13%), density (<8%), and permittivity (<1%) of the solutions are almost constant [38] and do not contribute much to the conductivity responses.

The cross-sensitivity of the modes towards viscosity *η* is studied using water solutions of glycerin as test liquid. Viscosities *η* of the solutions are varied from 1 to 1490 cP, while conductivity σ < 10^−3^ S/m, density ρ, and permittivity *ε_lq_* stay permanent within ±10% and give negligible contributions to acoustic responses for conductivity and viscosity.

Numerical data for *σ*, *η*, ρ, and *ε_lq_* of the solutions are determined from anhydrous solute weight per cent of the solution components [38]. Fabrication of the solutions is accomplished by mixing partial components in forced vibrator for about 5 min. The error in weight concentrations is about ±1%.

Cross-sensitivity of the samples towards temperature is studied by heating climatic camera UC-20CE from *T* = 0 to 55 °C with the step Δ*T* = 5 °C. The measurements are accomplished for amplitude response ΔS_12_ whose temperature variations without liquid in the cell are small (~0.1 dB) [39]. On the contrary, when liquid is present, the temperature response depends on liquid viscosity η at relevant temperature *T*. Extracting 1st data set from the 2nd, the temperature dependence of viscosity η(*T*) for a given liquid is measured. Precision of the measurements is ± 20%.

Results of the measurements are compared with numerical data calculated from simple formulas applied before to shear-horizontal acoustic modes in quartz plates loaded with conductive liquids [3]:

(2) $$\frac{\alpha }{\beta }=\frac{k^{2}}{2}\frac{\varepsilon_{pl}+\varepsilon_{0}}{\varepsilon_{pl}+\varepsilon_{lq}}\frac{\sigma \omega \left(\varepsilon_{pl}+\varepsilon_{lq}\right)}{\sigma^{2}+\omega^{2}{\left(\varepsilon_{pl}+\varepsilon_{lq}\right)}^{2}}\;$$

(3) $$\frac{\Delta v}{v_{0}}=-\frac{k^{2}}{2}\frac{\varepsilon_{pl}+\varepsilon_{0}}{\varepsilon_{pl}+\varepsilon_{lq}}\frac{\sigma^{2}}{\sigma^{2}+\omega^{2}{\left(\varepsilon_{pl}+\varepsilon_{lq}\right)}^{2}}\;$$

where *v*_o_ is the velocity of the wave at zero liquid conductivity, Δ*v* is the change in the velocity arising from liquid conductivity, α is the acoustoelectric attenuation, *β* is the wave number, *k*^2^ is the coupling constant of the wave, ω = 2π*f* is the wave frequency, and ε_lq_ and ε_pl_ are dielectric constants of the liquid and the plate, respectively. In particular, for quartz plates with small *k*^2^ = 3.2 × 10^−4^, acoustoelectric interaction at ω = 10^9^ s^−1^ produces quite measurable change in wave velocity or phase Δ*v*/*v*_o_ = Δϕ/ϕ ≈ 20 ppm, but negligible change in wave amplitude (acoustoelectric attenuation α = Δ*S*_12_*/L* ≈ 0.03 dB/mm). Because of that, the value of the attenuation for quartz plates was neither measured nor calculated [3]. However, substrates exhibiting higher coupling constant *k*^2^, such as lithium niobate, display grater attenuation, as shown in present paper.

Though formulas (2) and (3) directly relate acoustic (*v*, α) and electric (σ, ε_lq_) characteristics, they do not account for the difference in the modes of different orders, while the mode-liquid interaction should depend on electric potentials of the modes within conductive liquid. Therefore, results of the calculations may be compared with experimental data only qualitatively. In present paper, it is made for one of the modes detected in lithium niobate plate, as an example (*v_o_* = 14.093 m/s, *k*^2^ = 116 × 10^−4^, ω =10^9^ s^−1^).

## 3. Results and Discussion

Results obtained are presented in Figure 3, Figure 4, Figure 5, Figure 6, Figure 7, Figure 8, Figure 9 and Figure 10 and in Table 2 and Table 3.

**Figure 3:**
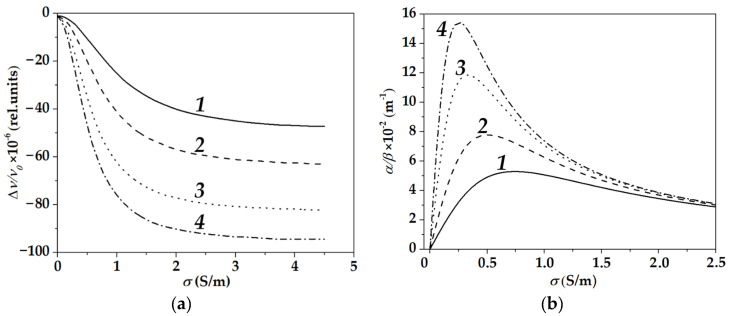
Changes in phase velocity Δv/v_o_ (**a**) and acoustoelectric attenuation *α/β* (**b**) versus liquid conductivity *σ* and dielectric permittivity *ε_lq_* calculated with formulas (2) and (3). Liquids: *ε_lq_*/*ε*_0_ = 79.3 (1), 52.5 (2), 32.9 (3), and 24.2 (4). Plate: 128°YX*-LiNbO*_3_, *h* = 500 μm, ε_pl_ = 34ε_0_. Wave: λ = 300 μm, *v* = 14.093 m/s, *k*^2^ = 116 × 10^−4^, ω =10^9^ s^−1^, displacements on the plate faces are {*u*_1_, *u*_2_, *u*_3_,} = {1, 0.36, 0.34}. β is the wave number.

According to formulas (2) and (3), both acoustic velocity *v* and attenuation α increase with conductivity σ of the liquid, while they decrease with liquid permittivity ε_lq_ (Figure 3). The changes produced by σ and ε_lq_ to *v* and α are comparable with each other.

In contrast to bulk and surface acoustic waves in piezoelectric semiconductors [40], acoustoelectric attenuation for acoustic plate modes and conductive liquids is not symmetric in respect to its maximum (Figure 3b): at the beginning, α increases with σ quickly, then approaches maximum at about σ ≈ 0.5 S/m, and finally, decreases slowly for σ > 0.5 S/m. The nature of the property is not clear yet, but very probable that it is originated from inhomogeneity of the propagation medium composed of a plate of one material and a liquid of another material and from inhomogeneity of the mode profiles through the depth of the propagation medium. Slow decrease in α after its maximum makes the range of wave sensitivity towards liquid conductivity σ wider.

**Figure 4:**
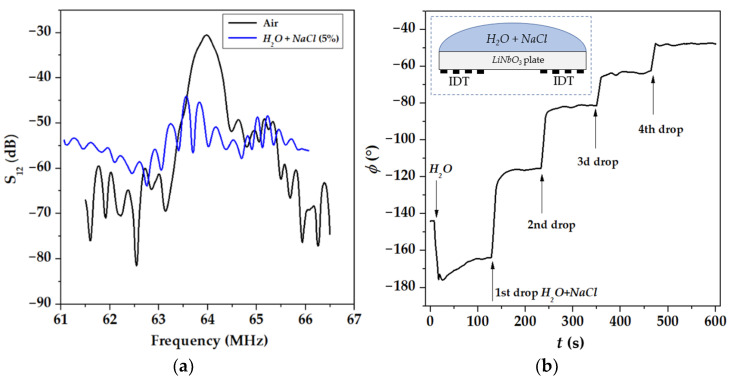
Insertion loss *S*_12_ (**a**) and phase *ϕ* (**b**) measured for AIR (without liquid) and conductive liquids (*H*_2_*O* + *NaCl* (5%)) with σ from 0 to 6 S/m *deposited over the whole propagation path* (30 mm). Plate: 128°YX-*LiNbO*_3_, *h* = 500 μm. Mode *f* = 63.9 MHz, λ = 200 μm. Test liquids: distilled water *H*_2_*O* (200 mg) and distilled water (200 mg) with drops of 0.9% *NaCl* (40 mg) in the water (arrows).

**Figure 5:**
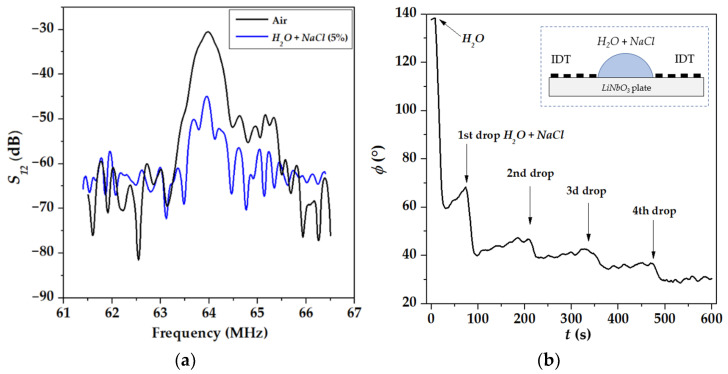
Insertion loss S_12_ (**a**) and phase ϕ (**b**) measured for AIR (without liquid) and conductive liquids (*H*_2_*O* + *NaCl* (5%)) with σ from 0 to 6 S/m *deposited only between transducers* (18 mm). Plate: 128°YX-*LiNbO*_3_, *h* = 500 μm. Mode *f* = 63.9 MΓц, λ = 200 μm. Test liquid: distilled water *H*_2_*O* (200 mg) and distilled water (200 mg) with drops of 0.9% NaCl (40 mg) in the water (arrows).

Figure 4 and Figure 5 demonstrate an important peculiarity of acoustic sensing conductive liquids: results of the measurements depend on the location of the liquid on the plate. When the liquid is over the whole propagation path, including two regions opposite to IDTs (Figure 4b, insert), insertion loss *S*_21_*(f)* are damaged by large electromagnetic leakage (horizontal level in Figure 4a) and phase ϕ is decreased with conductivity σ (Figure 4b). As far as ϕ~1/*v*, this behavior of the phase indicates that partial shorting of the plate face produced by the conductive liquid results in an increase in mode velocity *v* that is not right [7,41]. On the other hand, when the conductive liquid is only between transducers, outside the zone over them (Figure 4b), the leakage is much lower (15 dB below acoustic signal), insertion loss *S*_12_*(f)* is not damaged (Figure 4a), and phase ϕ is increased with conductivity σ (Figure 5), indicating a correct decrease in the mode velocity *v* in accordance with appropriate boundary conditions [7,41]. Following this peculiarity, all measurements of the present paper are accomplished with liquids localized, as shown in Figure 5.

**Figure 6:**
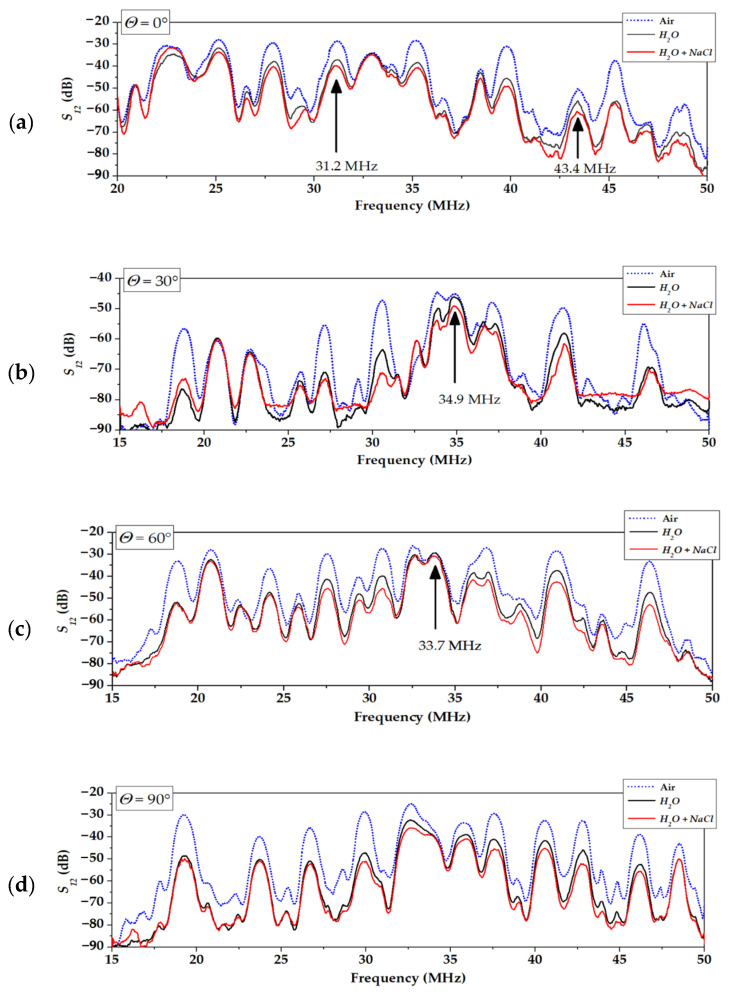
Insertion loss *S*_12_ of the delay lines with different propagation directions measured for various loadings on the path (L = 18 mm). Plate: 128°Y-*LiNbO*_3_, *h*/λ = 1.75 (*h* = 350 μm, λ = 200 μm). Propagation directions: *Θ* = 0° (**a**), 30° (**b**), 60° (**c**), and 90° (**d**) off X axis. Loadings: dashed lines correspond to case absence any liquid (AIR); black lines correspond to situation with presence of distilled water (*σ* = 0 S/m, 200 mg); red lines correspond to situation with presence of NaCl water solution (*σ* = 6 S/m, 200 mg). Bold arrows correspond to the modes with largest sensitivity towards liquid conductivity for each propagation direction.

Figure 6 shows typical spectra of acoustic plate modes in crystal plates with different propagation directions (angles *Θ*). For *Θ* = 0° (X axis) and *Θ* = 90° (perpendicular to X axis), all modes are “pure”: the beams of the modes are parallel to the wave vector and the power flow angles *Ψ_n_* are zero for all modes. 

**Figure 7:**
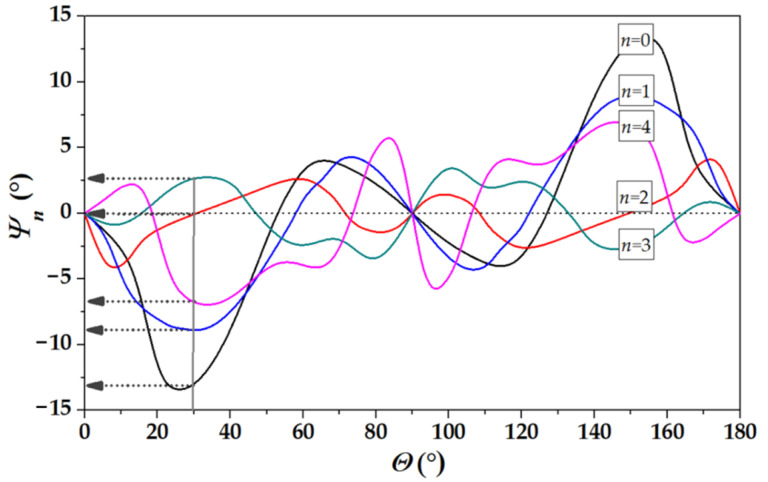
Orientation dependence of the power flow angles *Ψ_n_* for the first five modes propagating in 128°Y-*LiNbO*_3_ plate with normalized thickness *h*/λ = 1.75 (*h* = 350 μm, λ = 200 μm). The arrows indicate the angles *Ψ_n_* for direction Θ = 30° off X axis.

**Figure 8:**
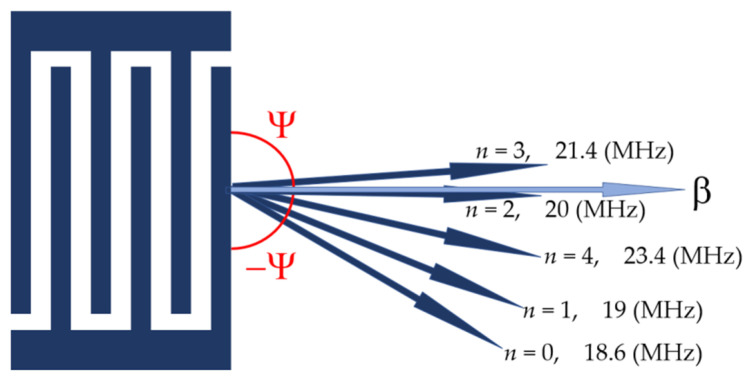
Schematic view of the fan-shaped power flow radiation from IDT on a plate surface for Lamb waves of different orders *n* (according to Table 2).

On the contrary, for propagation directions at *Θ* = 30° and *Θ* = 60° off the X axis, the beams are steering (“not pure” directions) and the power flow angles *Ψ_n_* are not zero for most modes. The values of the angles *Ψ_n_* are ranged from −13° to +13° depending on the mode order *n* (Figure 7, Table 2). As a result, each IDT radiates the modes not only at different frequencies (because *f_n_ = v_n_*/λ, where λ is the same IDT period for all modes, while *v_n_* is different for all modes), but at various angles *Ψ_n_* off the wave vector *β*, i.e., like a fan (Figure 8). In this case, some part of the beam’s energy misses output transducers, increasing the insertion loss *S*_12_ of the delay lines. For example, the mode *n* = 0 at Θ = 0° has *Ψ_n_* = 0° and *S*_12_(AIR) = 30 dB (Figure 6a), while the same mode at Θ = 30° has *Ψ_n_* = −13° and *S*_12_(AIR) = 56 dB (Figure 6b). Nevertheless, “not pure” modes may also be used for liquid sensing, in particular, for electric measurements. The modes have different responses Δϕ_12_ = ϕ_12_^Lq^ − ϕ_12_^H2O^ and Δ*S*_12_ = *S*_12_^Lq^ − *S*_12_^H2O^ for all propagation directions. The best of them for each direction is indicated in Figure 6 by bold arrows.

**Figure 9:**
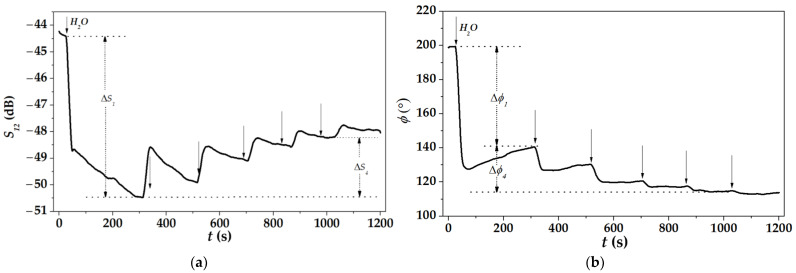
Time variations in the insertion loss *S*_12_ (mode amplitude) (**a**) and the phase *ϕ* (mode velocity) (**b**) versus step-by-step increase in liquid conductivity σ (vertical arrows). Plate: 128°YX-*LiNbO*_3_, *h* = 350 μm, λ = 200 μm. Mode: *f* = 34.9 MHz, propagation direction Θ = 30° off X axis. Reference liquid: distilled water (200 mg, *t* < 0 s). Test liquid: distilled water (200 mg) with drops of 0.9% *NaCl* + *H*_2_*O*, 40 mg each (vertical arrows, 0 < *t* < 1200 s).

Figure 9 shows typical behavior of the mode responses versus step-by-step increase in liquid conductivity. At the beginning (*t* < 0 s), when a cell contains distilled water as a reference liquid with σ = 0 S/m, both the amplitude and phase of mode are permanent (*S*_12_, *ϕ*_12_ = constants). When the first drop of conductive liquid is introduced into the water (first vertical arrow), the conductivity of the water-drop solution σ is increased from 0 to 2.8 S/m and both responses (Δ*S*_1_, Δ*ϕ*_1_) are changed immediately and remarkably: the amplitude of the mode is decreased (the attenuation is increased) and the phase of the mode is increased (the velocity is decreased). Next drops of the conductive liquid (other four vertical arrows from left to right) result in a further increase in conductivity σ of the liquid solution from 2.8 to 6.8 S/m and decreases in mode attenuation (Δ*S*_4_/4 in average) and velocity (Δ*ϕ*_4_/4 in average). The same behavior is inherent for all modes, plates, and propagation directions studied in the paper. The most attractive of them is presented in Table 3.

Table 3 shows that (i) the modes with enhanced electric sensitivity exist both for “pure” (*Θ* = 0°, 90°) and “not pure” (*Θ* = 30°, 60°) directions, (ii) the amplitude responses of the modes approach Δ*S*_1_ = 24 dB or 8.6 dB/(S/m) for the first drop and Δ*S*_4_/4 = 2.1 dB or 0.5 dB/(S/m) for the next four drops on average, (iii) relevant phase responses are as large as Δ***ϕ***_1_/4 = 300° or 107°/(S/m) and (Δ***ϕ***_4_/4) = 21.3° or 5.3°/(S/m), (iv) large amplitude responses may be accompanied by small phase responses and vice versa, though according to formulas (2) and (3), both acoustic characteristics should increase and decrease synchronously following the coupling constant *k*^2^ of the mode, and (iv) there are few modes with a good combination of all four sensing parameters (Δ*S*_1_, Δ*S*_4_/4, Δ***ϕ***_1_/4, and Δ***ϕ***_4_/4). Two of them are found along the Θ = 60° and Θ = 90° propagation directions (bold) in one and the same Y-*LiNbO*_3_ plate.

**Figure 10:**
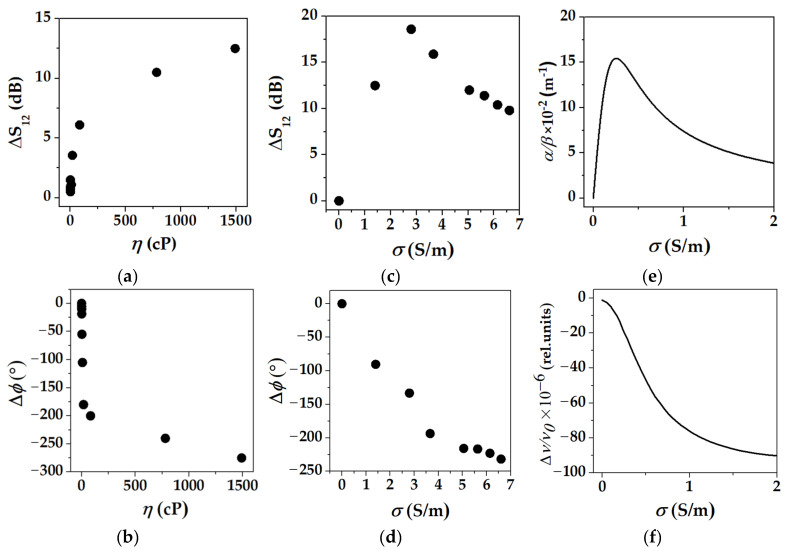
Calibration curves for one of the best modes towards liquid viscosity (**a**,**b**) and liquid conductivity (**c**,**d**) measured at 20 °C. Points are experimental data, solid lines (**e**,**f**) are numerical results calculated with formulas (2) and (3). Mode: *f* = 40.24 MHz, λ = 200 μm, *v* = 8.176 m/s, *k*^2^ = 110 × 10^−4^, surface displacements {*u*_1_, *u*_2_, *u*_3_,} = {1, 0.1, 0.5}. Plate: Y-LiNbO_3_, *h*/λ = 1.75, ε_pl_ = 34ε_0_. Propagation direction Θ = 60°. Liquids: water solutions of glycerin and *NaCl*, ε_lq_/ε_0_ = 79.3. Coated path L = 18 mm.

Figure 10 presents results of a mode calibration: (i) the curves for viscosity η (Figure 10a,b) are typical for waves vibrating at ultrasonic frequencies [3,42]. They are almost linear for small *η*, when liquid behaves as ideal (Newtonian), and saturates for large *η*, when liquid behaves as a solid, (ii) the curves for conductivity *σ* (Figure 10c,d) are also typical, in general [7,13]. Velocity of the mode decreases with σ monotonously, while acoustic attenuation jumps up initially, approaches maximum, and finally drops down slowly to zero for large *σ*, (iii) at the same time, the acoustoelectric attenuation has an important peculiarity. In contrast to surface and bulk acoustic waves propagating in piezoelectric semiconductors where the curve Δ*S*_12_(σ) is symmetric with respect to its maximum [40], the same curve for acoustic plate modes and conductive liquid is asymmetric (Figure 10c). It makes the range of detectable values wider: for phase response, the range of the measurements is restricted by about 5 S/m (Figure 10d), while for amplitude response, it is prolonged to 10–15 S/m (Figure 10c). This property is in qualitative agreement with numerical data calculated with formulas (2) and (3) (Figure 10e,f), but the nature of the property is not clear yet as already mentioned, (iv) because of the particular dependence on liquid conductivity σ (Figure 10d), the phase measurement provides a simple value of *σ* because each *ϕ* corresponds to a single *σ*; on the other hand, the phase response is temperature-dependent and demands careful thermal stabilization. Vice versa, the amplitude response Δ*S*_12_ is almost temperature-independent, but because of particular dependence on liquid conductivity *σ* (Figure 10c), it gives two different values *σ* for each Δ*S*_12_ at the same time and, thereby, demands additional measurements to avoid the wrong value. The maximal range of acoustic measurements is 0–10 S/m for phase output and 0–20 S/m for amplitude output. The volume of the test probe is about 100 μL in both cases, and (v) the sensitivity of the mode towards liquid conductivity (Figure 10c,d) is comparable with that for viscosity (Figure 10a,b), in general. Modes providing selective detection of conductivity, but not viscosity are not found in the paper.

## 4. Conclusions

Three peculiarities of acoustoelectric interaction between acoustic plate modes and conductive liquids deposited on piezoelectric plates are found in the paper: electromagnetic leakage between input and output transducers increases remarkably because of liquid conductivity, large increase in acoustoelectric attenuation may be accompanied by small decrease in phase velocity, and vice versa, the modes attenuation versus liquid conductivity is not symmetrical with respect to its maximum, making the range of detectable values wider. All peculiarities of the interaction are inherent for modes of different orders, polarizations, and beam steering. The sensitivity of the modes towards liquid conductivity is as large as 8.6 dB/(S/m) for amplitude and 107°/(S/m) for phase. The range of the measurements is restricted by about 10–15 S/m.

Because of anisotropy of the plates, there is beam steering for all modes. The magnitude of the steering depends on the mode order. The beam of different modes generated at various frequencies forms a fan around propagation direction on the surface of the plate. For some modes, the power flow angles are as large as ±13°.

Acoustic measurements of liquid conductivity through the phase of the waves are preferable when temperature of the liquid is constant. On the other hand, when the liquid temperature is varied somehow, the measurements of the wave amplitudes are more suited, though they give two different conductivity values for any acoustic amplitude. In this case, additional efforts to avoid a wrong value and to find the correct counterpart are required.

Responses of the modes towards liquid conductivity, viscosity, and dielectric permittivity found in the paper are comparable with one another in general.

## Figures and Tables

**Table 1:** The characteristics of the delay lines shown in Figure 1.

IDT Period, μm	IDT Aperture, μm	Number of Electrode Pairs in IDT	Face-to-Face Distance between Input and Output IDTs, μm	Distance L Coated with Liquid, μm	Plate Thickness h/λ
200	4900	20	24,000	18,000	1.75 or 2.5

**Table 2:** Phase velocities *v_n_*, power flow angles *Ψ_n_*, and own frequencies *f_n_* for the first five modes in 128°Y-*LiNbO*_3_ plate with normalized thickness *h/λ* = 1.75 (Θ = 30°).

*n*	*v_n_*, m/s	*Ψ_n_*, degr.	*f_n_*, MHz
0	3721.9	−13.1°	18.6
1	3804.95	−8.9°	19
2	4013.36	−0.1°	20
3	4272.11	2.7°	21.4
4	4678.26	−6.8°	23.4

**Table 3:** Acoustic plate modes with enhanced sensitivity towards liquid conductivity measured for different plates and propagation directions.

Plate	Θ, degr.	*h*/λ	*f*, MHz	Δ*S*_1_, dB	Δ*S*_4_/4, dB	Δ*ϕ*_1_, degr.	Δ*ϕ*_4_/4, degr.
128°Y-LNO	90°	2.5	36.55	11	0.6	60°	9.5°
128°Y-LNO	0°	1.75	31.18	7	0.8	64°	8.5°
128°Y-LNO	0°	1.75	43.4	6	0.23	44°	5.5°
128°Y-LNO	30°	1.75	34.9	6	0.65	59°	6.5°
41°Y-LNO	30°	1.75	35.925	10	0.8	67°	8.8°
41°Y-LNO	90°	1.75	33.62	15	1.5	**150**°	15°
Y-LNO	30°	1.75	40.42	9	0.8	40°	**21.3**°
Y-LNO	60°	1.75	40.24	**18**	**2**	**102**°	**32**°
Y-LNO	90°	1.75	38.72	4.6	0.6	37°	6.1°
Y-LNO	90°	1.75	52.48	**23**	1.4	**282**°	14°
Y-LNO	90°	1.75	58.46	**24**	**2.1**	**300**°	10°
36°Y-LTaO	0°	2.5	62.21	14	0.8	32°	5°
36°Y-LTaO	60°	2.5	31.19	8.7	0.75	82°	7.5°
36°Y-LTaO	90°	2.5	29	10	1	76°	13°
